# Supplementary material for: Identification of hub genes and transcription factor-miRNA-mRNA pathways in mice and human renal ischemia-reperfusion injury
Source: PeerJ. 2021 Oct 26;9:e12375. doi: 10.7717/peerj.12375 (PMC8555504; doi:10.7717/peerj.12375)

Before Normalization

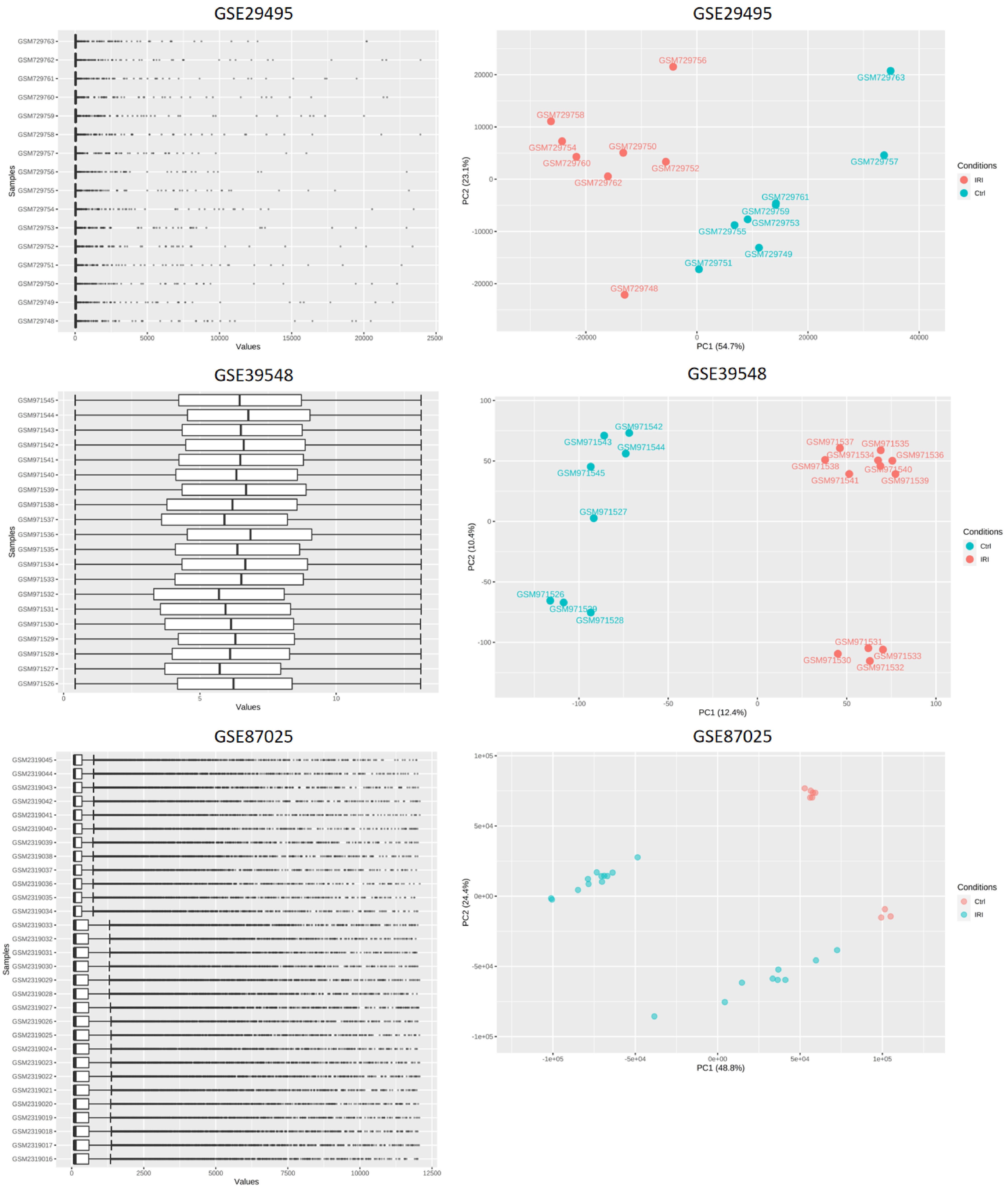

Before Normalization

GSE131288

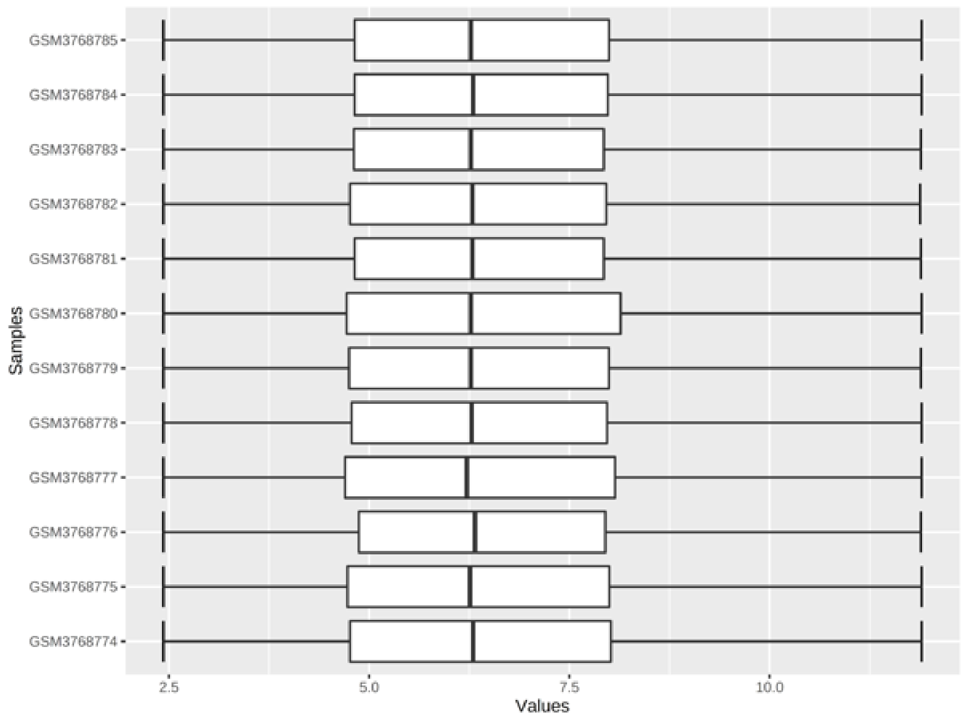

GSE131288

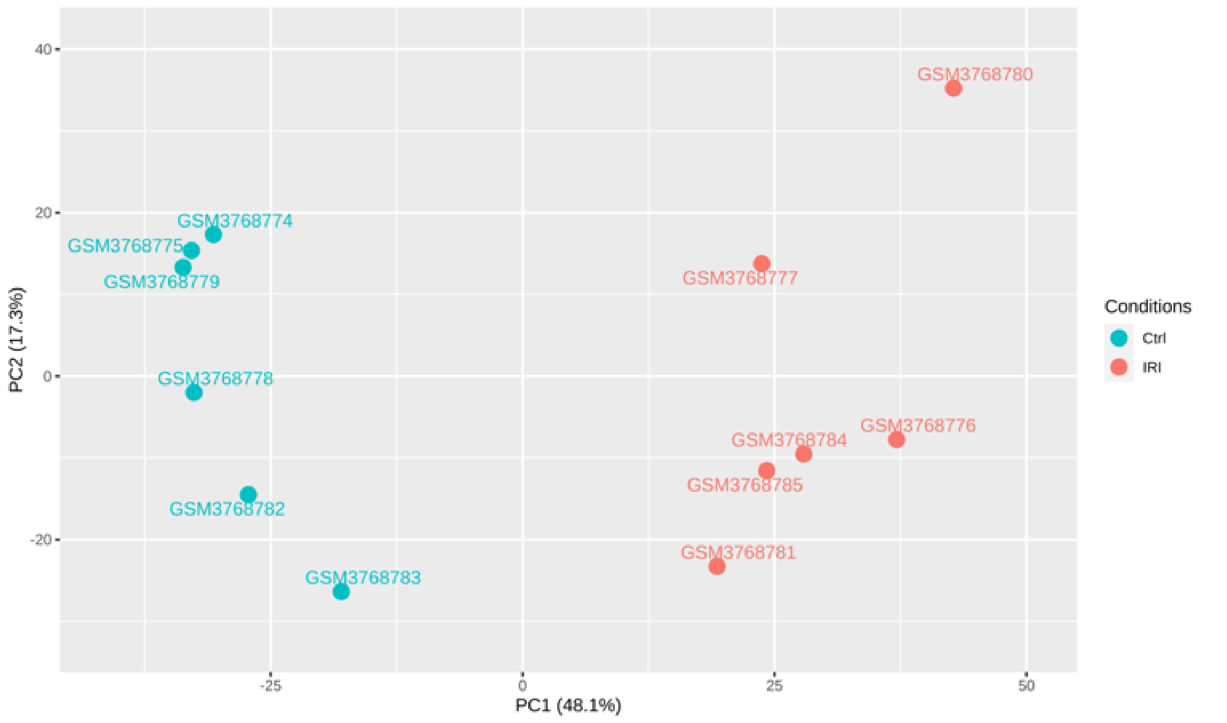

GSE71647

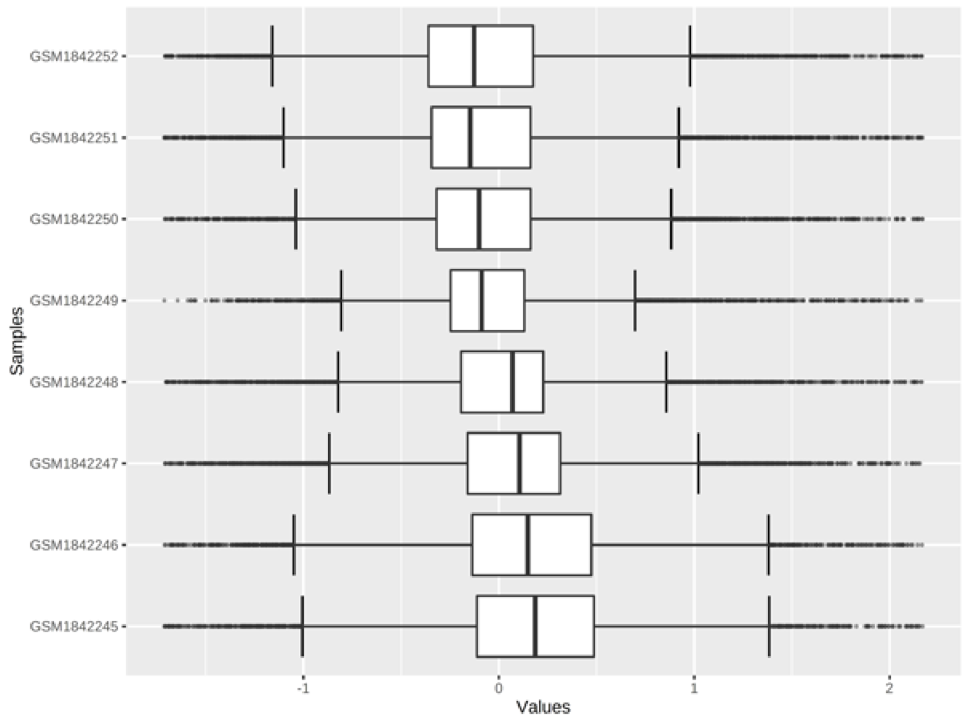

GSE71647

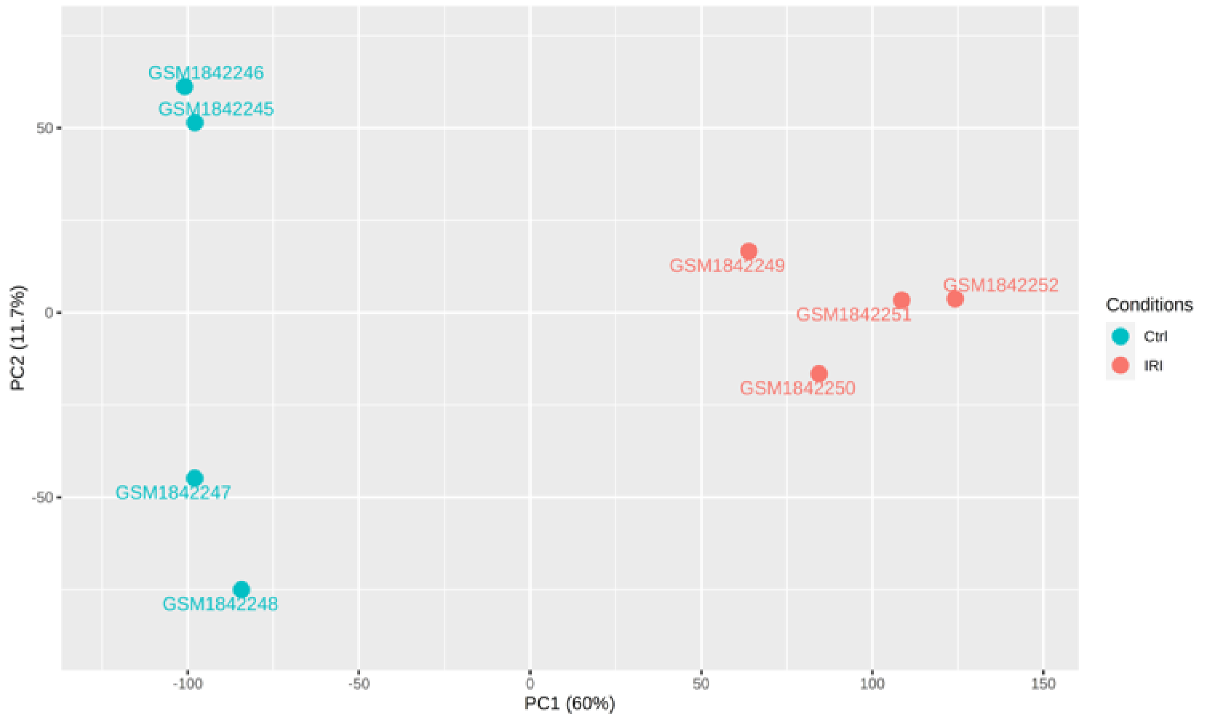

Before Normalization

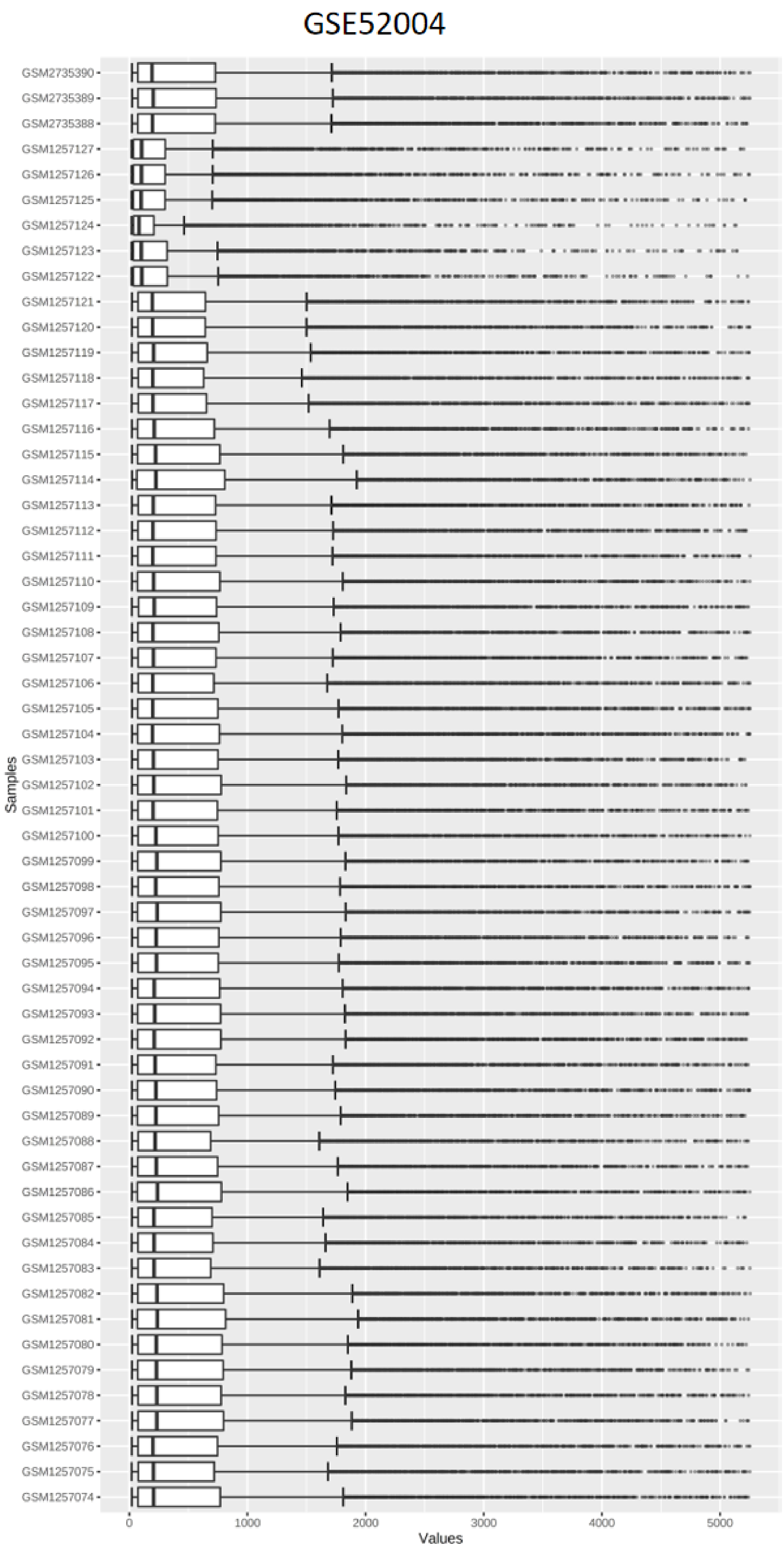

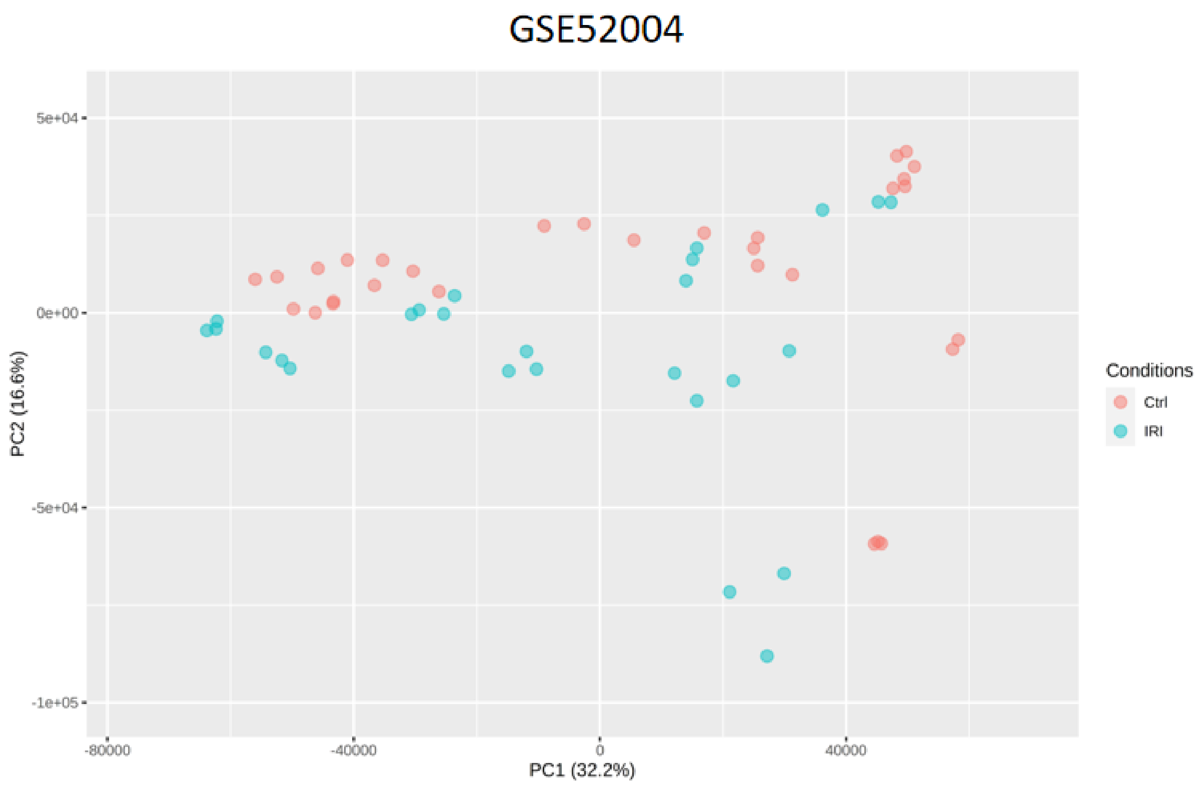

After Normalization

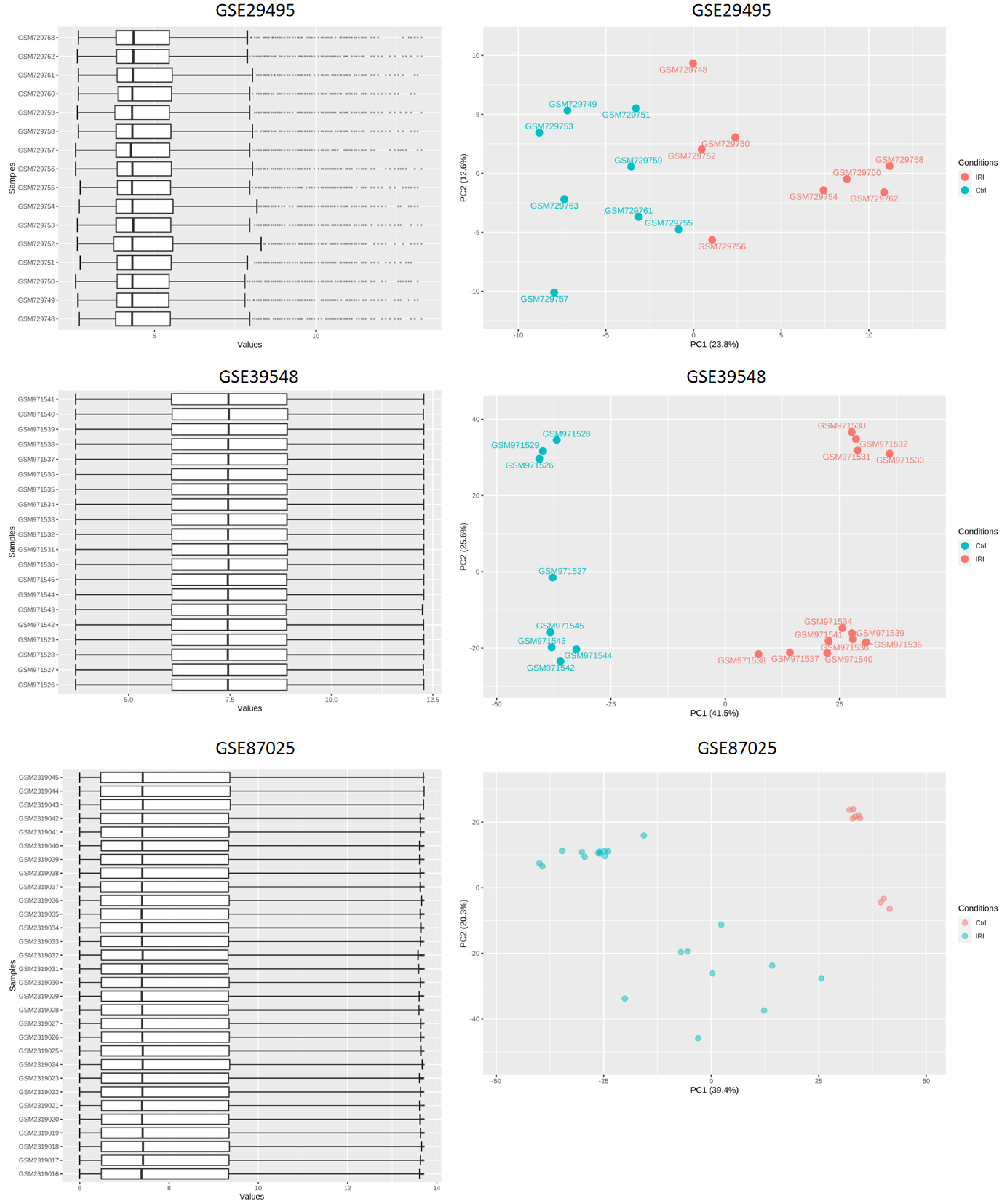

After Normalization

GSE131288

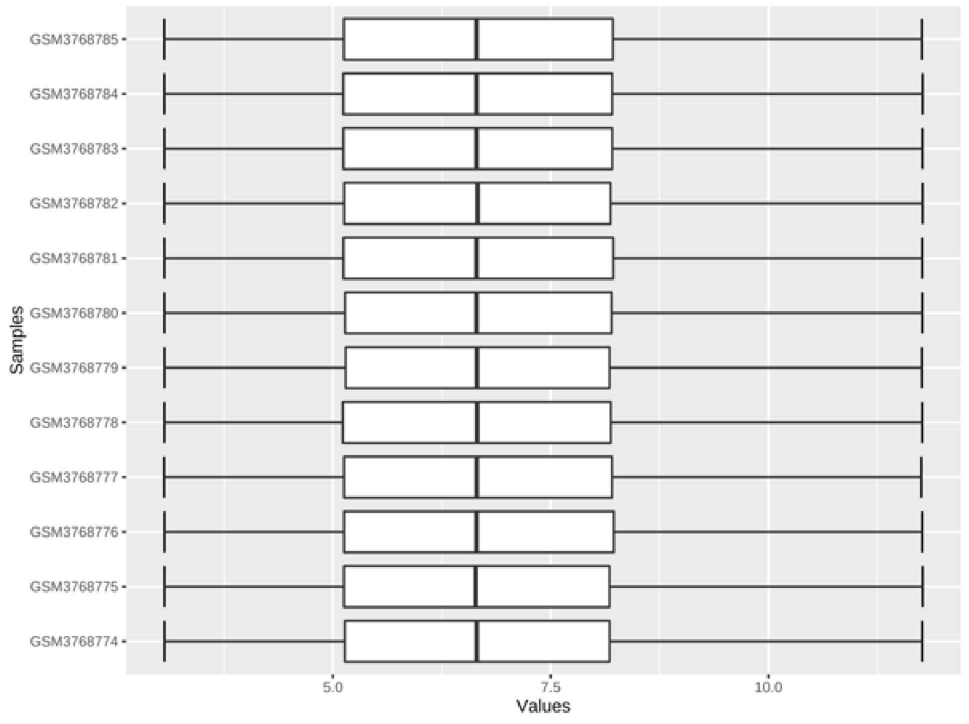

GSE131288

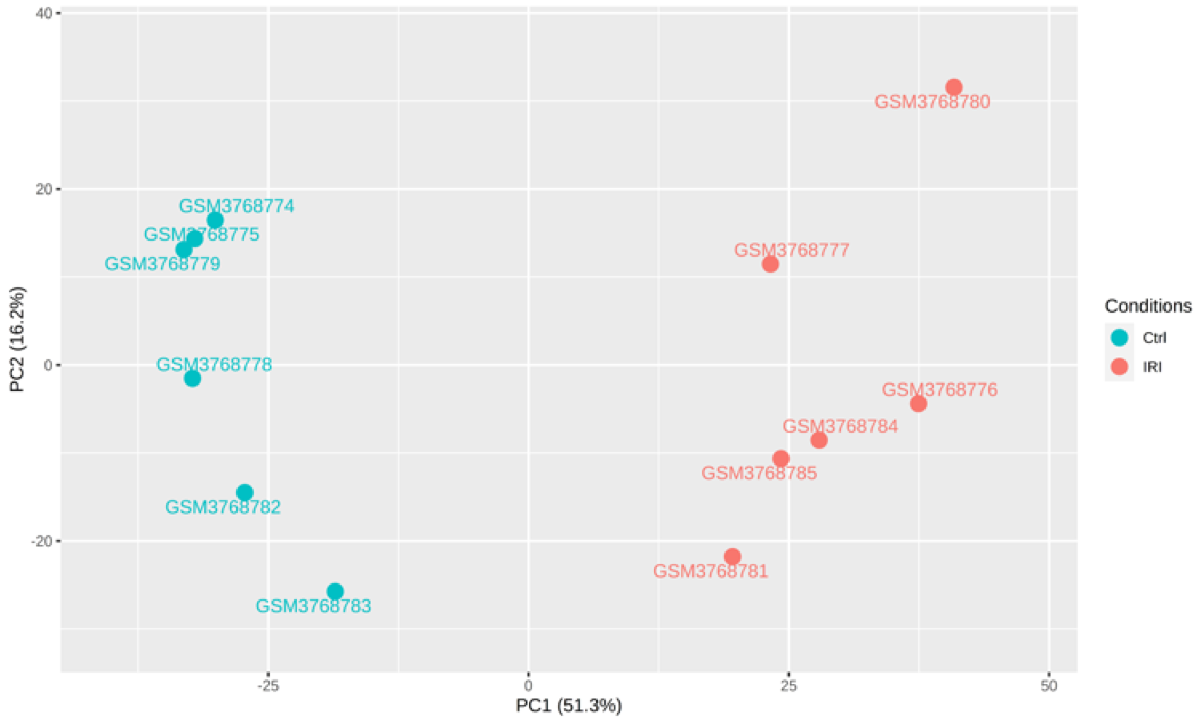

GSE71647

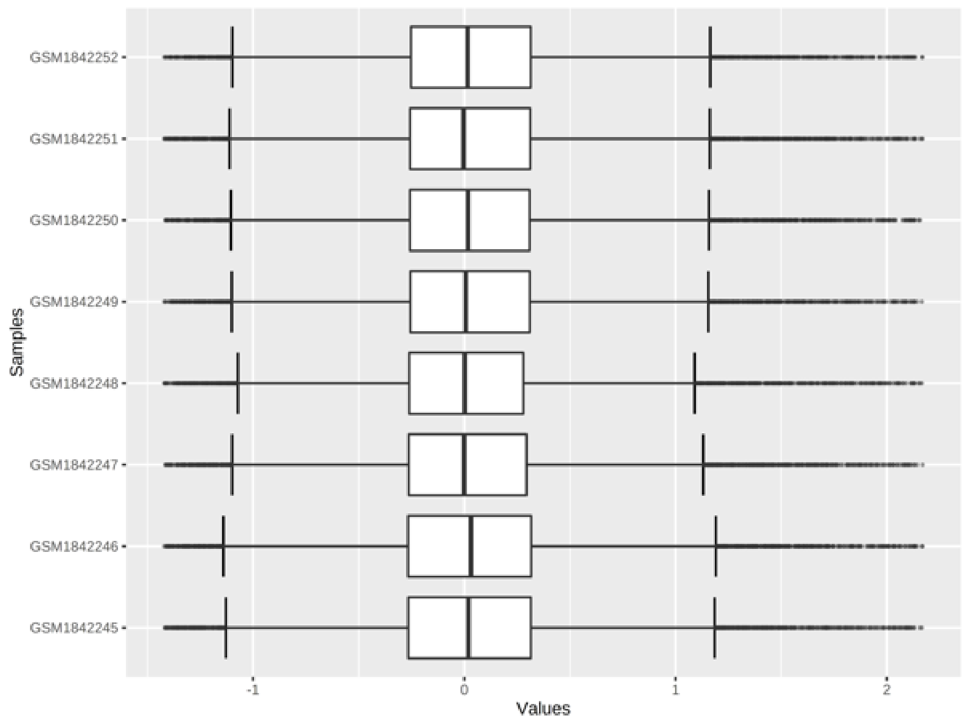

GSE71647

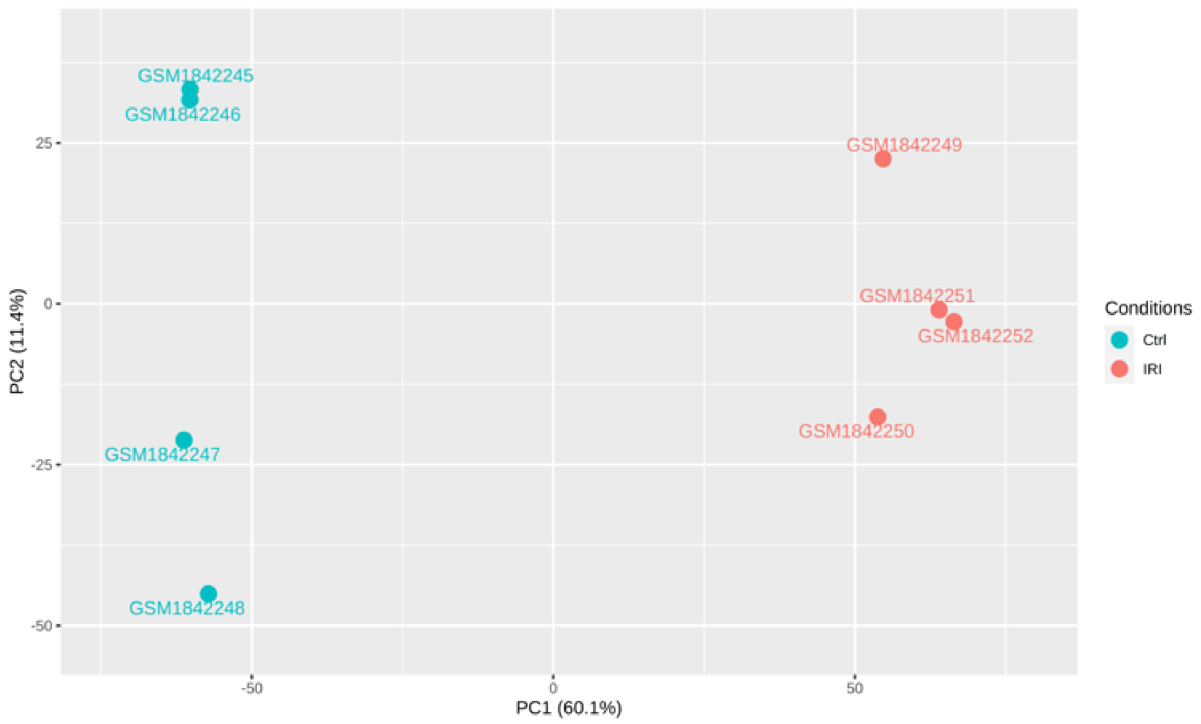

After Normalization

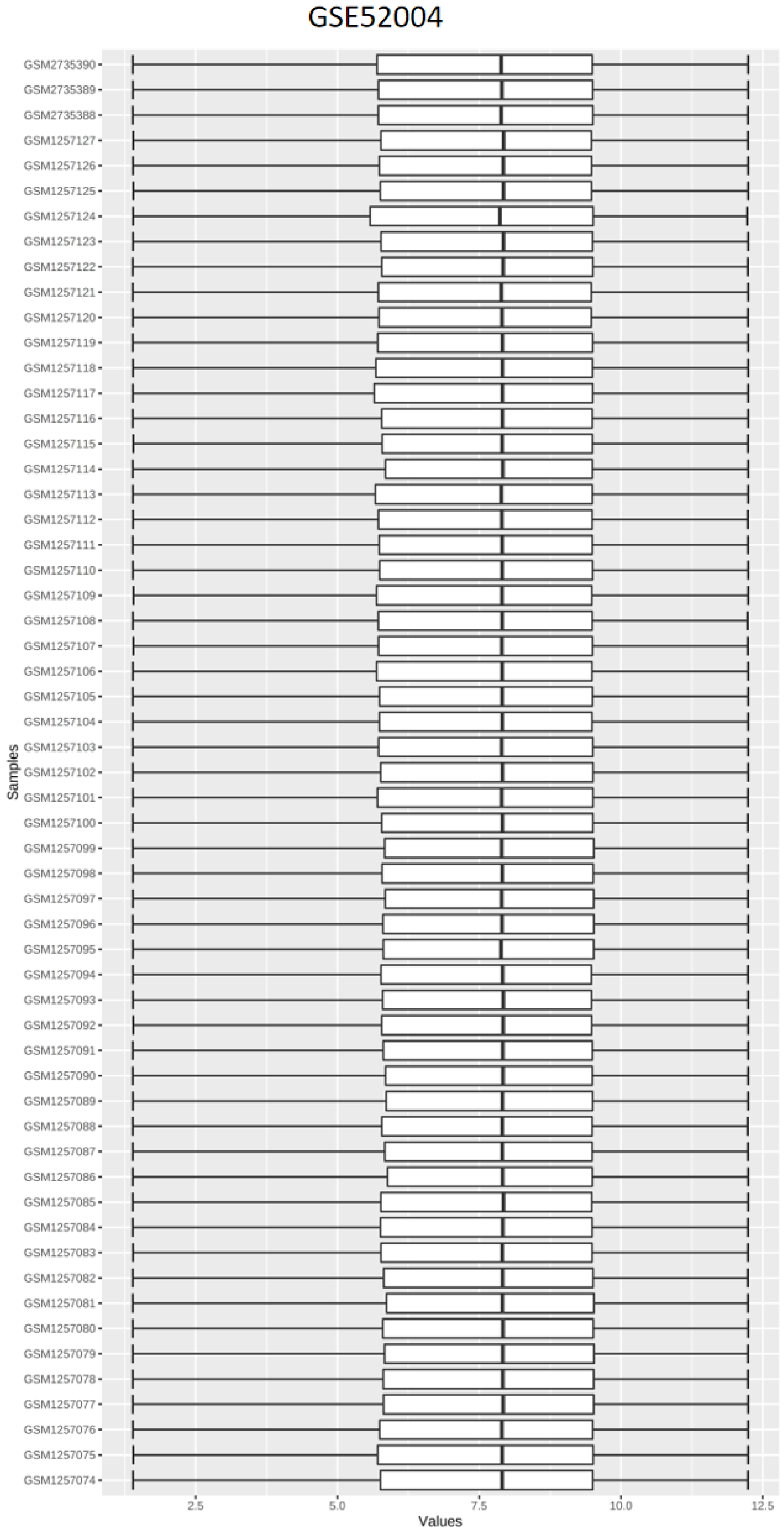

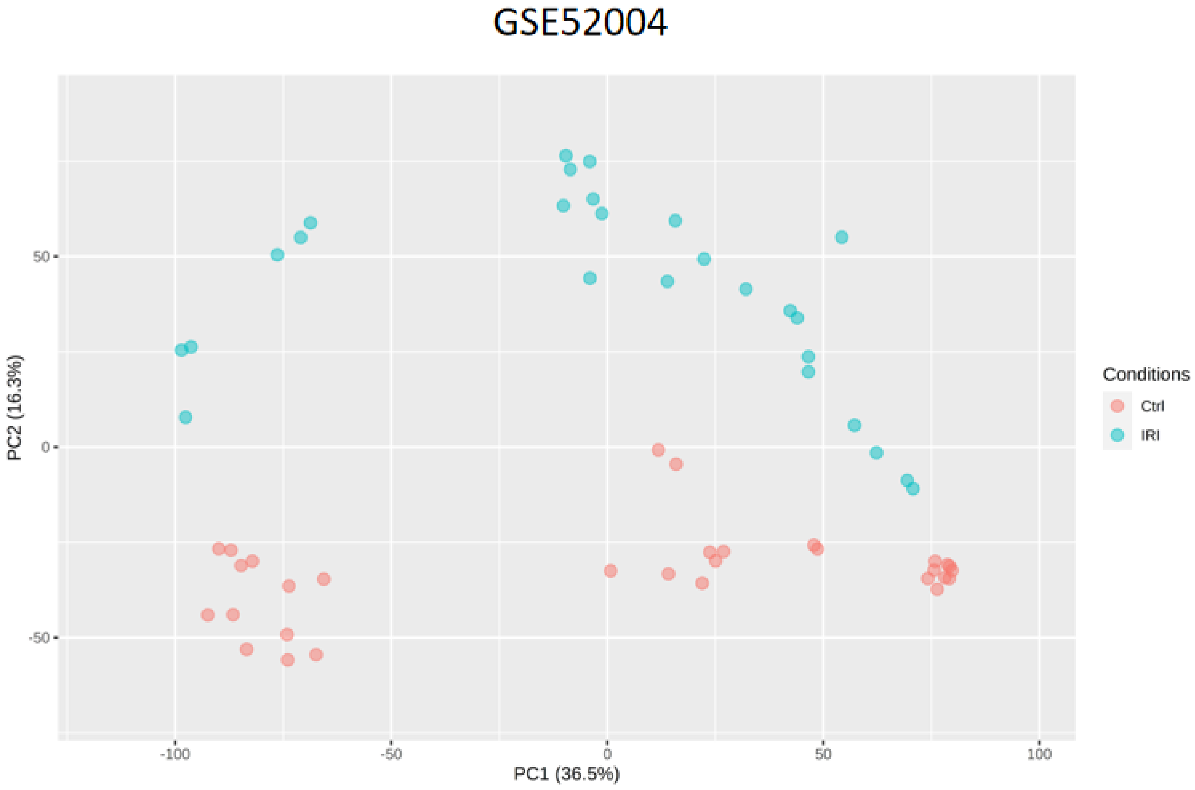

Supplement: Supplemental Information 2 [file peerj-09-12375-s002.pdf]
