# Supplementary figures and images for: Identification of hub genes and transcription factor-miRNA-mRNA pathways in mice and human renal ischemia-reperfusion injury
Source: PeerJ. 2021 Oct 26;9:e12375. doi: 10.7717/peerj.12375 (PMC8555504; doi:10.7717/peerj.12375)

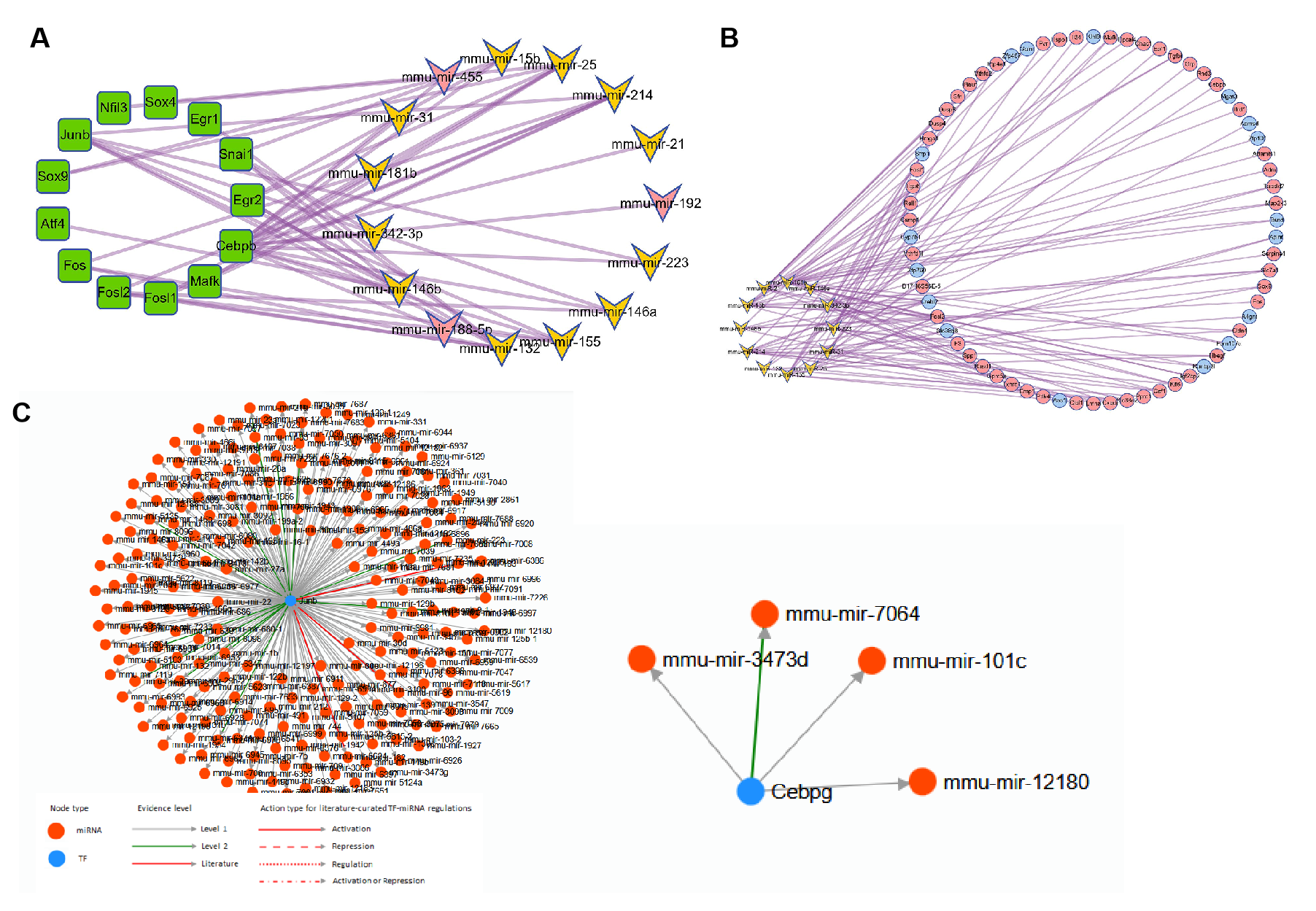

Supplement: Supplemental Information 6 — (A) The construction of TF-miRNA network and (B) miRNA-mRNA network. (C) Example of TF-mRNA from TransmiR v2.0. [file peerj-09-12375-s006.png]

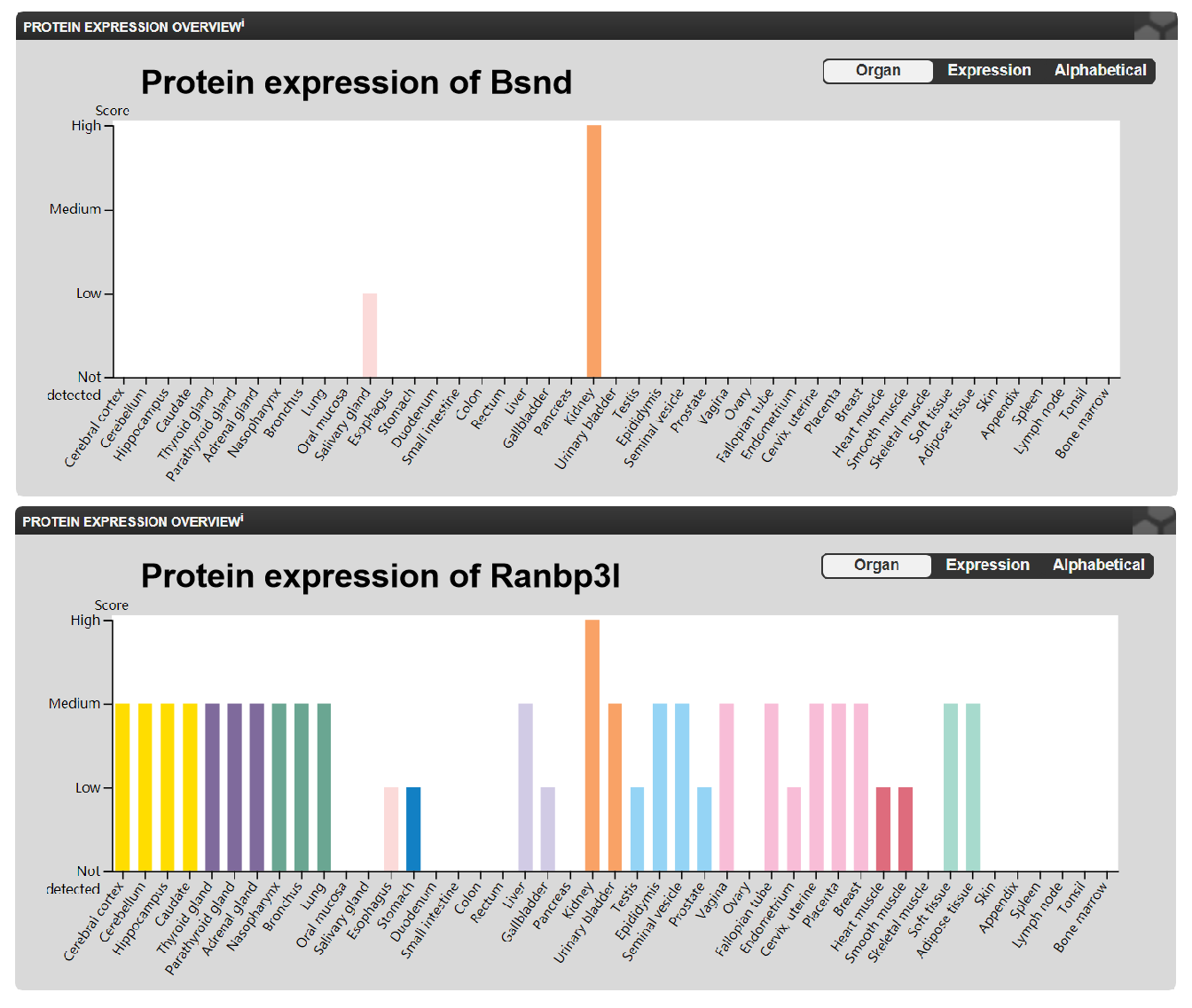

Supplement: Supplemental Information 8 [file peerj-09-12375-s008.png]
