# Supplementary material for: Identification of hub genes and transcription factor-miRNA-mRNA pathways in mice and human renal ischemia-reperfusion injury
Source: PeerJ. 2021 Oct 26;9:e12375. doi: 10.7717/peerj.12375 (PMC8555504; doi:10.7717/peerj.12375)
Supplement: Supplemental Information 9 [file peerj-09-12375-s009.docx]

| **RNA name** | **Forward sequences(5’-3’)** | **Reverse sequences(3’-5’)** |
| --- | --- | --- |
| Fos (homo） | CGTGCCAGACATGGACCTAT | CGGGGTAGGTGAAGACGAAG |
| Egr2 (homo） | AGTTTGCCCGGAGTGATGAG | CTGTTACTGCTGCACAGGGT |
| Junb (homo） | GTCAAAGCCCTGGACGATCT | TTGGTGTAAACGGGAGGTGG |
| Fosl2 (homo） | AACCTCGTCTTCACCTATCCT | AGTTCAAGGAGTCTGATGATTGG |
| Cebpb (homo) | CGACGAGTACAAGATCCGGC | TGCTTGAACAAGTTCCGCAG |
| Bsnd (homo） | GAGCTACAGTGAGGACCACC | TCACTCTCGTCTGAGCCCTT |
| Ranbp3l (homo） | GCATTCTCTTCCCAACCATCA | TGCTGTGTCATTCAGTCTCAAC |
| siRNA CEBPB | AGCACAGCGACGAGUACAAGA | UUGUACUCGUCGCUGUGCUTG |

**Supplementary file 9. Sequences of RNA and siRNA.**
